# A Giant Humeral Parosteal Lipoma

**DOI:** 10.7759/cureus.78401

**Published:** 2025-02-02

**Authors:** Vasco F Mendes, Joana Madeira, João S Silva, Amélia Estevão, Paulo Donato

**Affiliations:** 1 Radiology, Centro Hospitalar e Universitário de Coimbra, Coimbra, PRT; 2 Pathology, Centro Hospitalar e Universitário de Coimbra, Coimbra, PRT

**Keywords:** benign tumor shoulder, giant lipoma, parosteal lipoma, proximal humerus, rare benign tumor, shoulder tumor, soft tissue tumor

## Abstract

Parosteal lipomas are exceedingly rare and can pose diagnostic challenges due to their unusual imaging characteristics. Here, we report the case of a 70-year-old man who presented with a large painless shoulder mass that caused functional limitation. Imaging revealed a predominantly fatty lesion with an osseous excrescence and irregular cortical changes. Biopsy confirmed a parosteal lipoma, a rare benign fatty tumor arising directly on the bone cortex. Despite the atypical imaging features, which can mimic more aggressive tumors, surgical excision was performed due to the size and functional impact. After three years, the patient showed no recurrence and improved shoulder range of motion.

## Introduction

Parosteal lipomas are rare benign tumors of fatty tissue that arise directly on the bone cortex. These lesions have a juxtacortical location and are characterized by an osseous excrescence attached to the cortical surface [1,2]. Despite their benign nature, parosteal lipomas can be mistaken for malignant or aggressive tumors due to their size and the potential for misdiagnosis as atypical lipomatous tumors or high-grade liposarcomas [1]. We report the case of a man in his 70s with a large painless lesion on his left shoulder, which had slowly grown over the past 20 years and resulted in a limited range of motion.

## Case presentation

A man in his 70s presented to the hospital with a large painless lesion on his left shoulder that caused functional limitation. Physical examination revealed a large mass with a firm consistency, and the patient reported that it had been growing slowly over the past 20 years.

**Figure 1:**
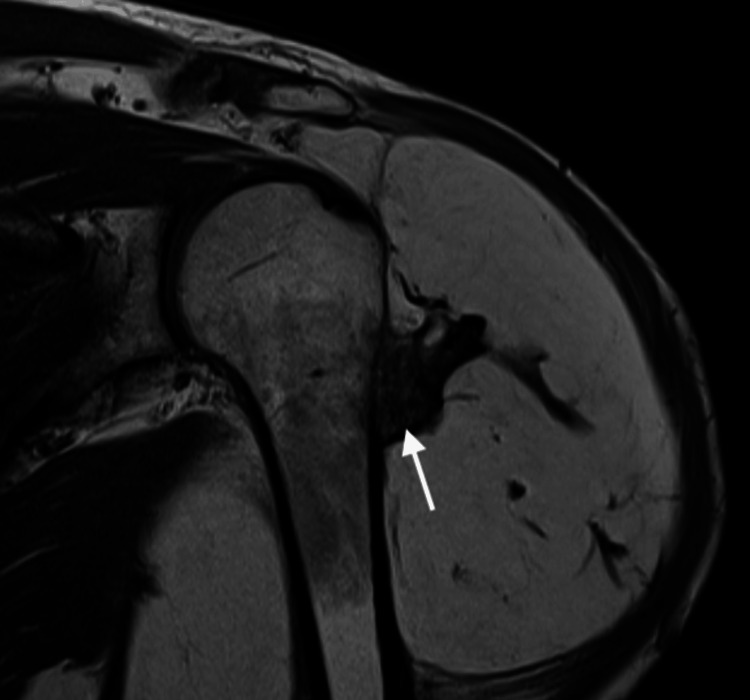
Coronal T1-weighted MRI. A hyperintense lesion adjacent to the left humerus, consistent with fatty tissue, can be observed. The lesion includes a bony prominence adjacent to the cortical surface (arrow), with no apparent continuity with the humeral bone marrow.

**Figure 2:**
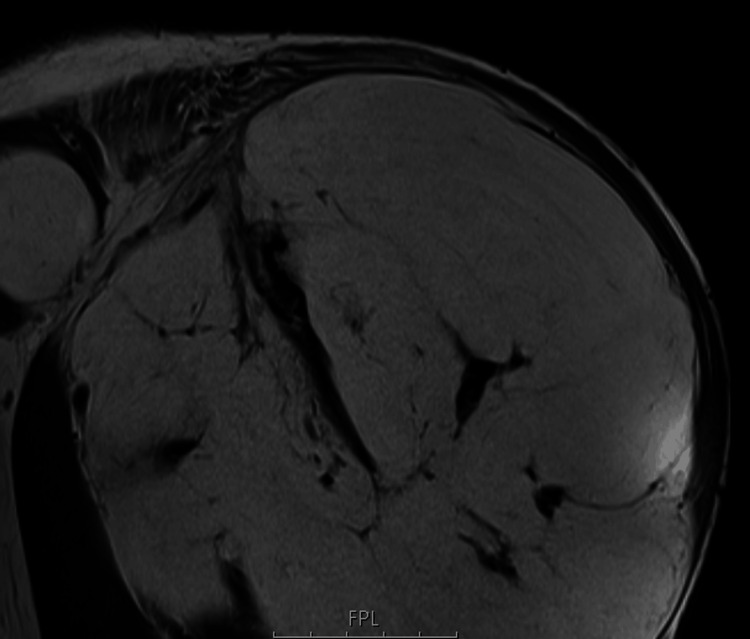
Sagittal T1-weighted MRI. The lesion exhibits predominantly hyperintense signal on T1 (fatty tissue), with scattered areas of hypointensity (bony tissue) observed throughout.

**Figure 3:**
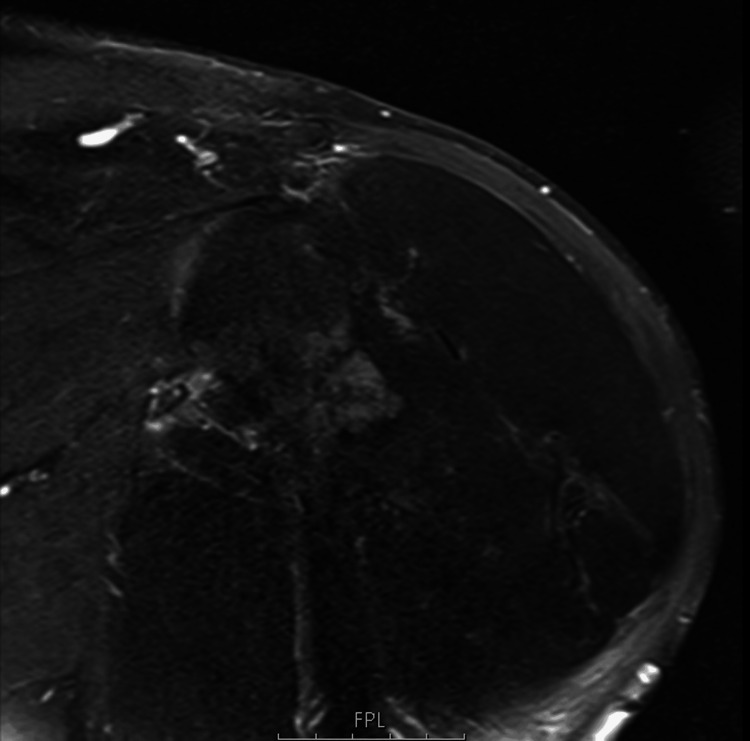
Coronal T2-weighted fat-saturated MRI. The lesion demonstrates low signal on T2 fat saturation, supporting the hypothesis of adipose content.

MRI revealed a large lesion (20 × 17 × 15 cm) composed predominantly of fat, with some areas of hypointensity on T1 and T2 sequences. Additionally, the adjacent humerus displayed cortical irregularity but no cortical disruption (Figures 1-3).

**Figure 4:**
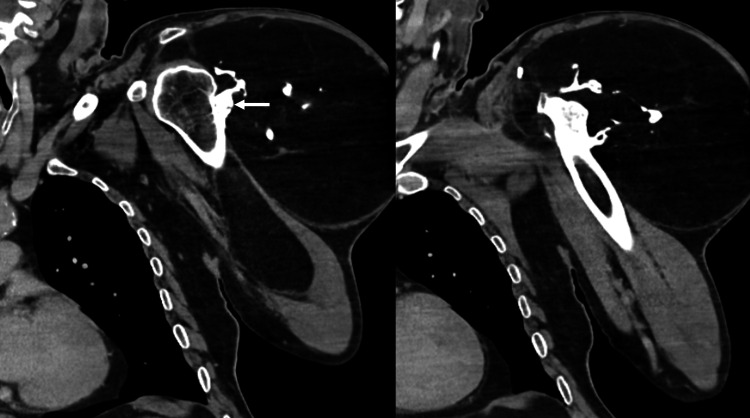
Coronal CT. A large lipomatous lesion adjacent to the left humerus is visible. An irregular osseous excrescence (arrow) is noted, lacking continuity with the humeral bone marrow. Additionally, multiple other foci of ossification within the lesion are observed.

**Figure 5:**
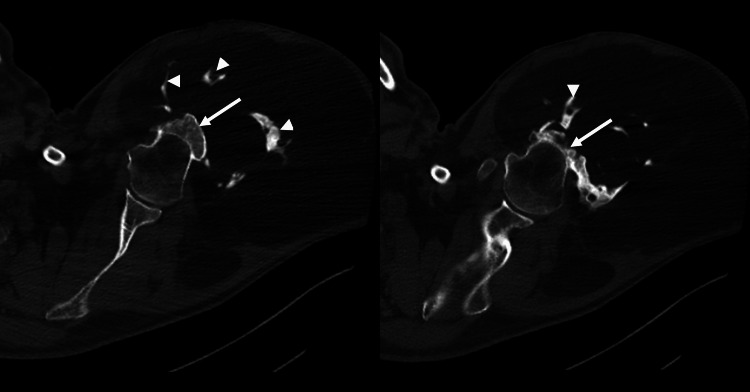
Axial bone window CT. A large lipomatous lesion adjacent to the left humerus is visible. An irregular osseous excrescence (arrow) is noted, lacking continuity with the humeral bone marrow. Additionally, multiple other foci of ossification within the lesion are observed (arrowheads).

**Figure 6:**
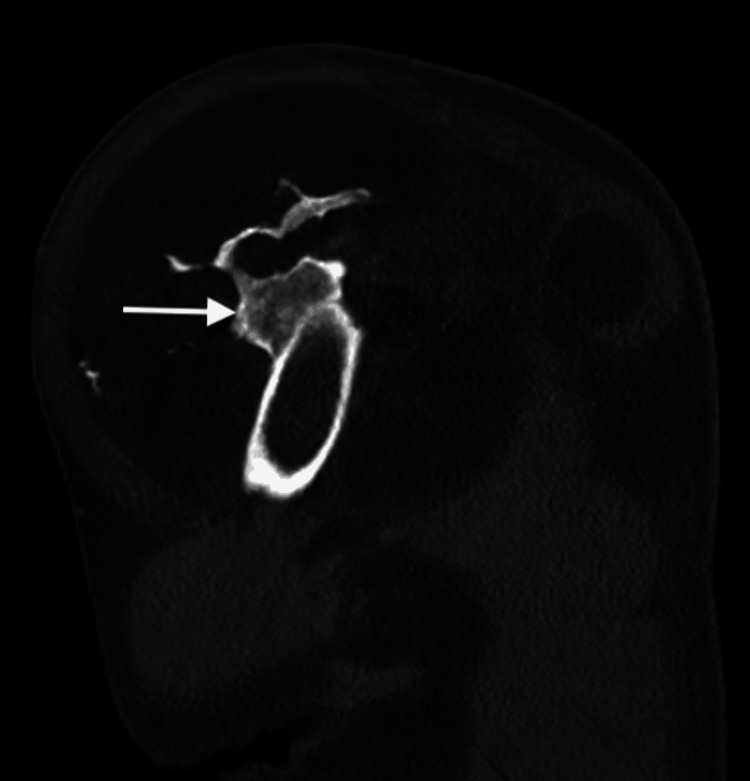
Sagittal bone window CT. A large lipomatous lesion adjacent to the left humerus is visible. An irregular osseous excrescence (arrow) is noted, lacking continuity with the humeral bone marrow.

CT imaging confirmed that most of the mass consisted of adipose tissue. It also revealed an exostotic component originating from the mid-third of the humerus and linear foci of ossification (Figures 4-6).

**Figure 7:**
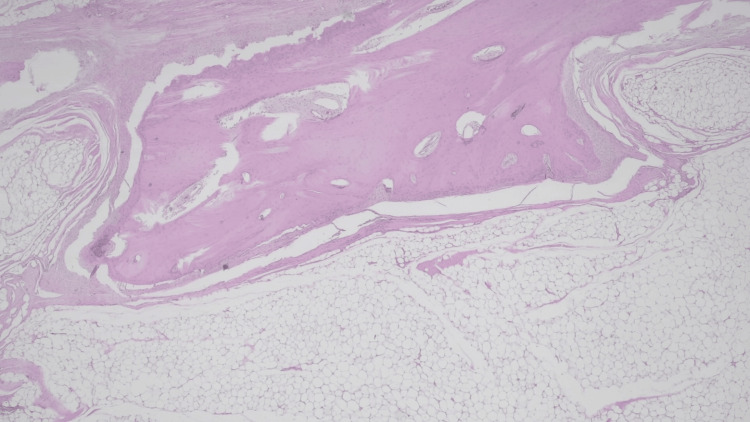
Histopathological image of hematoxylin and eosin staining at 20× magnification. The image shows an area of proliferation of mature polyhedral adipocytes with a single central vacuole, displacing the pyknotic nucleus to the periphery. The adipocytes are interspersed with fibrous septa of varying thickness, containing spindle-shaped cells without atypia. Additionally, a region of mature trabecular bone is visible.

**Figure 8:**
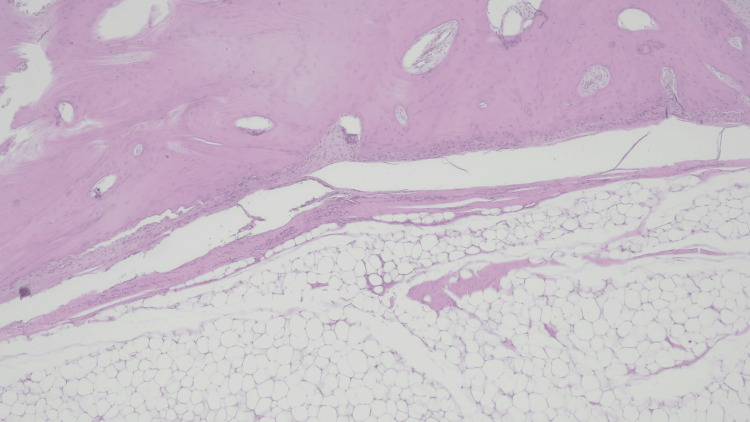
Histopathological image of hematoxylin and eosin staining at 40× magnification. The image shows an area of proliferation of mature polyhedral adipocytes with a single central vacuole, displacing the pyknotic nucleus to the periphery. The adipocytes are interspersed with fibrous septa of varying thickness, containing spindle-shaped cells without atypia. Additionally, a region of mature trabecular bone is visible.

An ultrasound-guided biopsy demonstrated adipose tissue without atypia. Given the lesion’s size and associated functional limitation, surgical excision was performed. Histopathological analysis showed mature, polyhedral adipocytes with a single central vacuole, displacing the pyknotic nucleus to the periphery, interspersed with fibrous septa of variable thickness containing spindle cells without atypia, as well as areas of mature trabecular bone (Figures 7, 8).

After three years of follow-up, there were no signs of recurrence, and the patient experienced an improved range of motion in his left shoulder.

## Discussion

The detection of musculoskeletal lipomatous lesions on imaging studies is a common finding in clinical practice. Most of these lesions are benign, such as soft tissue lipomas, which account for approximately 50% of all soft tissue tumors. However, a wide spectrum of rarer lipomatous lesions also exists [1].

Parosteal lipomas are exceedingly rare lipomatous neoplasms, constituting only 0.2% of all lipomas and fewer than 0.1% of all bone tumors. These benign fatty tumors arise directly from the bone cortex and typically occur in individuals aged 40 to 60 years [2].

Parosteal lipomas are located juxtacortically and are characterized by an osseous excrescence attached to the cortical surface [1-3]. Non-lipomatous elements within the fatty portion of the mass, as well as foci of internal mineralization distinct from the osseous excrescence, have been described. Adjacent bone commonly exhibits cortical thickening or periostitis, and, in rare cases, bone marrow edema may be present [2]. Osseous excrescences, when observed, can be distinguished from osteochondromas by their lack of continuity with the marrow space of the underlying bone and the absence of a cartilaginous cap [3]. These bony excrescences may show uptake on fluorodeoxyglucose positron emission tomography/computed tomography [2].

Despite their benign nature, parosteal lipomas may mimic malignant or aggressive tumors due to their size and imaging characteristics, potentially leading to misdiagnoses as atypical lipomatous tumors or high-grade liposarcomas [1-4]. The associated osseous stalk and internal mineralization can also be misinterpreted as malignant osteoid matrix production or aggressive periosteal changes. The rarity of this entity, combined with its atypical imaging features, may lead to unnecessary invasive procedures or surgical interventions [2,4].

Surgical resection is typically reserved for symptomatic patients, such as those with limited range of motion, nerve entrapment, or aesthetic concerns [4].

## Conclusions

Parosteal lipomas are extremely rare benign fatty tumors that originate from the bone cortex. Despite their benign nature, they may be mistaken for malignant or aggressive tumors due to their size and the presence of calcifications. Therefore, the diagnosis of parosteal lipoma should be considered when evaluating lipomatous lesions with an osseous excrescence, especially in cases of slow growth, to avoid confusion with malignant pathology that could result in inappropriate treatment or unnecessary, potentially mutilating, surgical procedures.
